# How many ways are there to die? Identification of ICU death typologies using cluster analysis

**DOI:** 10.1186/cc13225

**Published:** 2014-03-17

**Authors:** L Reineck, A Barnato, R Arnold, D Angus, J Kahn

**Affiliations:** 1 University of Pittsburgh, PA, USA

## Introduction

Although avoidance of death is a key goal of critical care, so too is provision of high-quality end-of-life care when life-prolonging therapy is not desired. One often-used measure of ICU performance is risk-adjusted mortality, yet this measure treats all deaths equally and thus is flawed. In this work, we identify different death typologies using cluster analysis, with the ultimate goal of more narrowly targeted ICU quality improvement measures and efforts.

## Methods

We performed k-means cluster analysis in 2,047 ICU decedents admitted to a university medical center in 2011/12. Variables for the analysis included ICU length of stay (LOS), mechanical ventilation, tracheostomy, gastrostomy tube insertion, dialysis, enteral or parenteral nutrition, and cardiopulmonary resuscitation (CPR).

## Results

Four clusters were identified. Short ICU LOS and low life- sustaining therapy utilization but relatively frequent CPR characterized Cluster 1. Intermediate ICU LOS and moderate life-sustaining therapy utilization characterized Clusters 2 and 3. Prolonged ICU LOS and high life-sustaining therapy utilization (except CPR) characterized Cluster 4. Age and severity of illness decreased across clusters with the oldest, sickest patients in Cluster 1 and the youngest, least sick patients in Cluster 4. See Table 1.

**Table 1:** 

	Cluster 1 (*n *= 353)	Cluster 2 (*n *= 755)	Cluster 3 (*n *= 667)	Cluster 4 (*n *= 272)
ICU LOS, mean ± SD (days)	0.5 ± 0.2	2.0 ± 0.8	7.2 ± 2.8	25.6 ± 15.2
Mechanical ventilation	282 (79.9%)	631 (83.6%)	604 (90.6%)	269 (98.9%)
Tracheostomy	3 (0.9%)	13 (1.7%)	14 (2.1%)	127 (46.7%)
CPR	20 (5.7%)	19 (2.5%)	16 (2.4%)	8 (2.9%)

## Conclusion

Cluster analysis can identify unique typologies of death within ICUs. This approach may be a novel method to more specifically target ICU efforts to reduce mortality and improve the quality of death.

